# Assessment of Electromyographic Changes in Masseter and Temporalis Muscles for Patients Undergoing Lower Third Molar Surgery: A Prospective Study

**DOI:** 10.7759/cureus.59489

**Published:** 2024-05-01

**Authors:** Soorumsetty Ruthvik, Melvin George, Suresh Venugopalan, Vaishnavi Rajaraman, Santhosh P Kumar, Gidean A Sundaram

**Affiliations:** 1 Oral and Maxillofacial Surgery, Saveetha Dental College and Hospitals, Saveetha Institute of Medical and Technical Sciences, Saveetha University, Chennai, IND; 2 Prosthodontics and Implantology, Saveetha Dental College and Hospitals, Saveetha Institute of Medical and Technical Sciences, Saveetha University, Chennai, IND

**Keywords:** mouth opening, innovative technique, novel method, electrical activity, trismus, third molar surgery, temporalis, masseter, electromyography (emg)

## Abstract

Introduction

Lower third molar impaction surgery is one of the most common minor oral surgical procedures done. Trismus has been one of the most common and disturbing postoperative sequelae for patients. The study aimed to evaluate the electrical activity of the masseter and temporalis muscles after mandibular third molar surgery.

Materials and methods

The research was conducted at Saveetha Dental College and hospitals in the Department of Oral and Maxillofacial Surgery. The study consisted of 20 individuals. The EMG (electromyography) activities of both masseter muscles in each patient were measured before the tooth extraction surgery, postoperatively after 72 hours, and after seven days. The inter-incisal distance was also measured at similar follow-up intervals. Data were analyzed using IBM Corp. Released 2015. IBM SPSS Statistics for Windows, Version 23.0. Armonk, NY: IBM Corp., with p-values less than 0.05 considered statistically significant. The Mann-Whitney U test was used for the comparison of electrical activity between masseter and temporalis on both the operated and non-operated sides during preoperative, postoperative, 72-hour, and postoperative seven-day periods.

Results

It has been found that the electrical activity of the temporalis is higher than that of the masseter muscle measured at all the intervals of the follow-up period, with statistically significant values (p=0.001). It was noted that all the patients have reduced mouth opening when compared with preoperative (mean mouth opening = 45.6 mm), postoperative 72 hours (mean mouth opening = 31.2 mm), and postoperative seven days (mean mouth opening =35.6 mm). When a comparison was done between temporalis and masseter, the masseter took longer to return to pre-operative electrical activity, which might also imply that for prolonged trismus seen in patients after lower third molar surgery, it is the masseter that is affected and needs recovery for trismus to be resolved.

Conclusion

Based on the results obtained, it can be concluded that there was a reduction in the electrical activity of both the masseter and temporalis post-third molar impaction surgery. It was also found that there was a reduction in mouth opening in patients who underwent lower third molar extraction surgery. Masseter muscle took longer to return to its preoperative electrical activity than temporalis muscle, implying that targeted therapies to accelerate the healing of masseter muscle may prevent prolonged trismus in patients who undergo lower third molar impaction surgery.

## Introduction

Surgical removal of an impacted mandibular third molar is considered one of the most common types of minor oral surgical procedures [1]. Mandibular third molars, in their attempt to erupt, trigger a lot of pathological conditions, leading to eventual surgical extraction [2]. Impaction, despite being the most common procedure, is also associated with many complications, mostly occurring postoperatively, like postoperative swelling, pain, and trismus. Intraoperative complications include uncontrollable hemorrhage, neurological damage, and mandibular fractures (rare) [3]. Complications are common, but measures taken to evaluate the severity of the complications and the measures that have been taken to overcome them make all the difference [4].

After surgical removal of the mandibular third molar, the most common complaint noticed by the patients is restricted mouth opening, or, in other words, trismus. The most commonly used technique to assess trismus is to measure preoperative and postoperative inter-incisal distance. A more precise, objective scale that determines individual muscle function is necessary to assess trismus [5]. One such test for objective assessment is electromyography (EMG) assessment of the masticatory muscle function. One of the main clinical applications of EMG is to identify any abnormalities in muscle function and muscle activity [6]. In oral and maxillofacial surgery, EMG has been mainly used for the analysis of masticatory muscle dynamics and the electrical activity of the same. Electromyography (EMG) is a technique in which there is registration of electrical activity that is generated by the muscle cells when they are stimulated either electrically or neurologically [7]. These signals are measured on the skin surface using surface electrodes as the sum of the electrical activity of that particular muscle fiber.

Many studies have proved that trismus and reduced masticatory muscle electrical activity are interrelated [8]. The most common reason for trismus seen in patients who visit the dental hospital is the post-surgical removal of impacted teeth. Trismus is not only related to the relative injury done to the respective muscle fibers but also to the time taken for recovery of those muscle fibers, and the recovery rates of each masticatory muscle fiber are at different rates. Based on the available literature and also comparing the preoperative muscle activity, we can assess the time taken for recovery of the muscle, and based on that data, we can detect the muscle that is recovering late and is causing prolonged trismus in patients [9].

In the present study, the EMG activities of the masseter and temporalis muscles were assessed preoperatively and postoperatively after surgical removal of impacted lower third molars. The EMG (electromyography) activity was analyzed using the surface electrodes. This study aimed to evaluate the changes in electrical activity in the masseter and temporalis muscles on the operated and non-operated sides in the same patients who underwent lower third molar impaction surgery.

## Materials and methods

Study design 

This was a prospective comparative study that was done in the Department of Oral and Maxillofacial Surgery at Saveetha Dental College and Hospital. The total period of the study was four months, starting from June 2023 to October 2023. The total sample size was determined as per the G* power calculation, with the study requiring a total sample size of 20 patients. A prior proforma with informed consent was obtained from all the participants in the study. The study got approval from our Institutional Human Ethical Committee (IHEC/SDC/OMFS-2106/23/056). All the participants were randomly assigned to the study using a simple random sampling technique. Surgical removal of impacted teeth was done under aseptic conditions under local anesthesia by the same surgeon, and assessments were done by the same principal investigator for all the participants.

Inclusion and exclusion criteria

**Figure 1:**
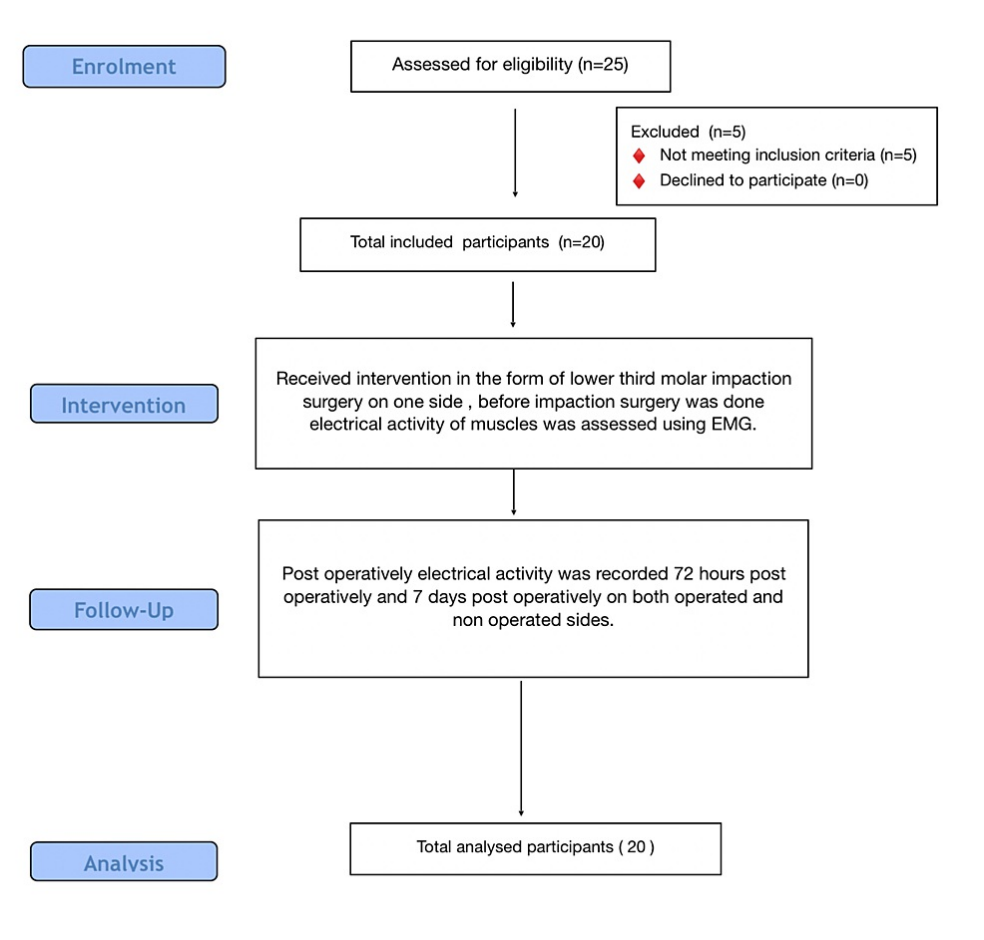
Methodology Flow Chart.

All the patients, irrespective of sex, with ages ranging from 20 to 40 years, except those who wanted prophylactic removal of impacted lower third molars, were included in the study. Patients with muscle-related systemic diseases such as myasthenia gravis, patients with temporomandibular joint disease, patients who had undergone multiple tooth extractions, patients with facial asymmetries, and patients with American Society of Anesthesiologists (ASA) classifications of III or IV were excluded from the study. Figure 1 shows the methodology flow chart.

Surgical procedure 

**Figure 2:**
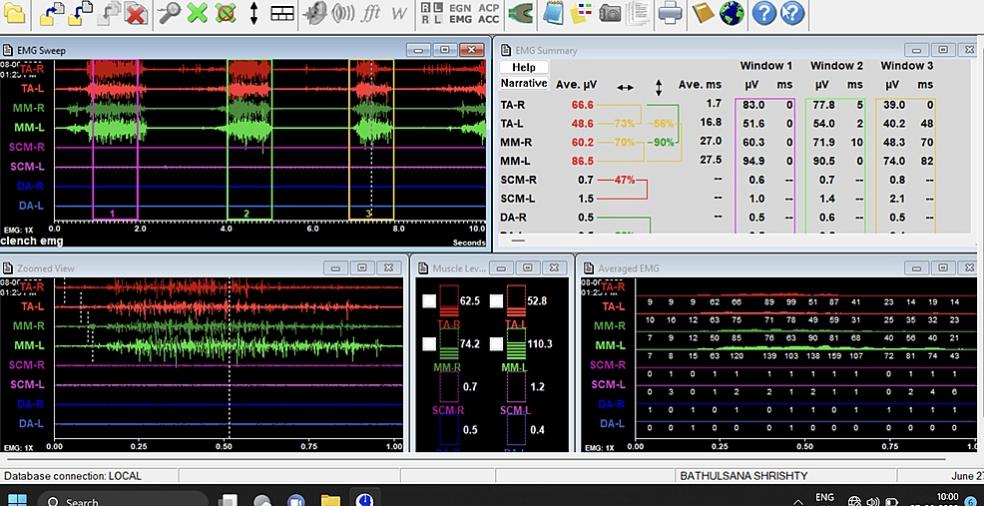
Electromyography of Temporalis and Masseter muscles.

Under sterile aseptic conditions, standard scrubbing was performed both intraorally and extraorally. Local anesthesia with a 1:80,000 dilution of adrenaline was given as an inferior alveolar nerve block on the side planned to be operated on in modified wards. An incision was placed, a full-thickness mucoperiosteal flap was elevated, and bone guttering was done using the Moore and Gillbe collar technique. The EMG activities of both the masseter and temporalis muscles in each patient were measured before the tooth extraction operation, postoperatively after 72 hours, and postoperatively after seven days. Chewing gum was provided to each patient, and the patients were asked to chew the gum on both sides. Dynamic EMG activity was recorded over 30 seconds of chewing the gum. A comparison of the masseter and temporalis muscles’ EMG activity postoperatively was done with the values on the non-surgical side and the values obtained preoperatively from both sides (Figure 2). Maximum mouth opening was also measured by measuring the interincisal distance from the dental midline. 

Follow-up

The parameters that were assessed in the study are the electrical activity of the masseter and temporalis muscles and the maximum mouth opening at pre- and post-impaction surgery of the third molar. Electrical activity was measured using surface electrodes, while maximum mouth opening was measured using a vernier caliber preoperatively, 72 hours postoperatively, and seven days postoperatively after lower third molar impaction surgery.

Statistical analysis

The data was collected and analyzed with means and standard deviations with a 95% confidence interval using IBM Corp. Released 2015. IBM SPSS Statistics for Windows, Version 23.0. Armonk, NY: IBM Corp. The Mann-Whitney U test was used for comparison of electrical activity between the masseter and temporalis on both the operated and non-operated sides during the preoperative, postoperative, 72-hour, and seven-day periods.

## Results

Our study population consisted of nine males and 11 females, with a mean age of 27 ± 5.5 years. For all patients in this study, maximum mouth opening was recorded preoperatively, 72 hours postoperatively, and seven days postoperatively. Electrical activity of two groups of muscles, i.e., the masseter and temporalis, was recorded on both the operated and non-operated sides at specific and regular follow-up time intervals.

It was found that the mean maximum mouth opening among our study participants preoperatively was 45.69 mm, which after the third molar impaction surgery had decreased to 35.65 mm, 72 hours postoperatively, and 39.19 mm 7 days postoperatively (Table 1). 

**Table 1:** Mean Maximum Mouth Opening at Regular Follow-Up Intervals Among the Study Participants.

	preoperative	72 hours postoperative	7 days postoperative
mean of maximum mouth opening	45.69 mm	35.65 mm	39.19 mm

A comparison of muscle activity using EMG between the masseter and temporalis muscle groups on the non-operated side at preoperative, postoperative, 72 hours, and seven days was done. It was found that median electromyographic values were statistically significantly higher in the temporalis group than the masseter group at preoperative (p = 0.001), postoperative 72 hours (p = 0.001), and postoperative seven days (p = 0.001), respectively (Table 2). 

**Table 2:** Comparison of Muscle Activity Between the Masseter and Temporalis Muscle Groups on the Operated Side at Different Time Intervals. N: number; IQR: interquartile range; **Statistically significant using Mann-Whitney U test; mV: millivolts

Operated	Muscle group	N	Median	IQR	p-value
Preoperative	Masseter Group	20	1079 mV	1069.75 - 1082.75 mV	P = 0.001**
	Temporalis Group	20	1434.5 mV	1416 - 1445.75 mV	
Postoperative 72 hours	Masseter Group	20	692.5 mV	671.5 - 717.75 mV	P = 0.001**
	Temporalis Group	20	1216 mV	1200.25 - 1227.75 mV	
Postoperative 7 days	Masseter Group	20	872 mV	845.25 - 927.75 mV	P = 0.001**
	Temporalis Group	20	1377 mV	1361 - 1388.5 mV	

A comparison of muscle activity using EMG between the masseter and temporalis muscle groups on the operated side at preoperative, postoperative, 72 hours, and seven days was done. It was found that median electromyographic values were statistically significantly higher in the temporalis group than the masseter group at preoperative (p = 0.001), postoperative 72 hours (p = 0.001), and postoperative seven days (p = 0.001), respectively (Table 3). 

**Table 3:** Comparison of Muscle Activity Between Operated and Non-Operated Sides for the Masseter Group at Different Time Intervals. N: number; IQR: Interquartile range; NS: Not significant; **Statistically significant using the Mann-Whitney U test; mV: Millivolts

Masseter Group	Side	N	Median	IQR	p-value
Preoperative	Operated	20	1079 mV	1069.75 - 1082.75 mV	P = 0.157
	Non-operated	20	1056 mV	1025 - 1111.75 mV	NS
Postoperative 72 hours	Operated	20	692.5 mV	671.5 - 717.75 mV	P = 0.001**
	Non-operated	20	807.5 mV	798.25 - 829 mV	
Postoperative 7 days	Operated	20	872 mV	845.25 - 927.75 mV	P = 0.001**
	Non-operated	20	1030 mV	1020 - 1038.25 mV	

**Figure 3:**
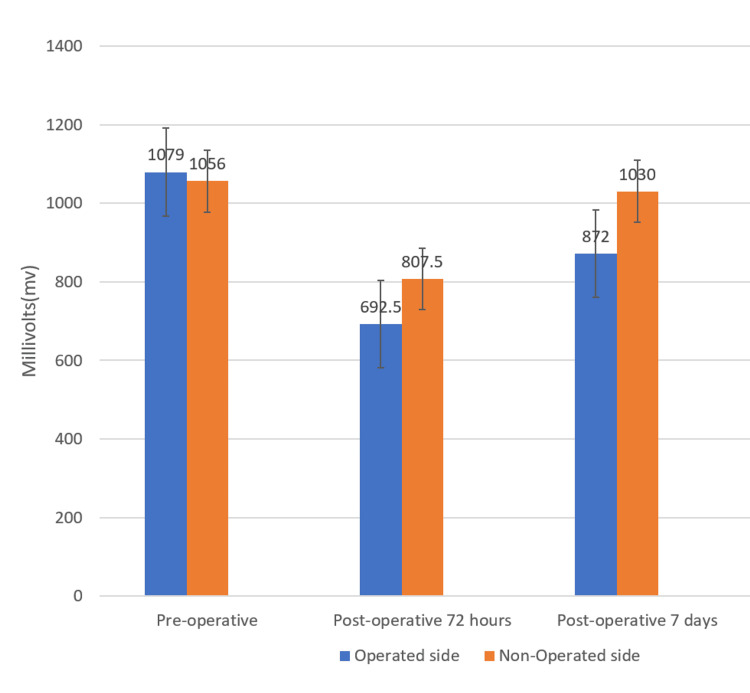
Median Muscle Activity Values Between Operated and Non-Operated Sides for the Masseter Group at Preoperative, Postoperative 72 Hours, and Postoperative 7 Days.

A comparison of median values between operated and non-operated sides for the Masseter group at preoperative, postoperative, 72 hours, and seven days was done. It was found that median electromyographic values were statistically significantly higher on the non-operated side than the operated side in the Masseter group at postoperative 72 hours (p = 0.001) and postoperative 7 days (p = 0.001), respectively. The electromyographic values were similar on both the operated and non-operated sides preoperatively (p = 0.157) (Figure 3 and Table 4).

**Table 4:** Comparison of Muscle Activity Between Operated and Non-Operated Sides for the Temporalis Group at Different Time Intervals N: number; IQR: interquartile range; **Statistically significant using the Mann-Whitney U test; mV: millivolts.

Temporalis Group	Side	N	Median	IQR	P-value
Preoperative	Operated	20	1434.5 mV	1069.75 – 1082.75 mV	P = 0.001**
	Non-operated	20	1454 mV	1440 – 1472.75 mV	
Postoperative 72 hours	Operated	20	1216 mV	1200.75 – 1227.75 mV	P = 0.001**
	Non-operated	20	1358 mV	1330 – 1378 mV	
Postoperative 7 days	Operated	20	1337 mV	1361 – 1388.5 mV	P = 0.001**
	Non-operated	20	1415 mV	1409.25 – 1419.75 mV	

**Figure 4:**
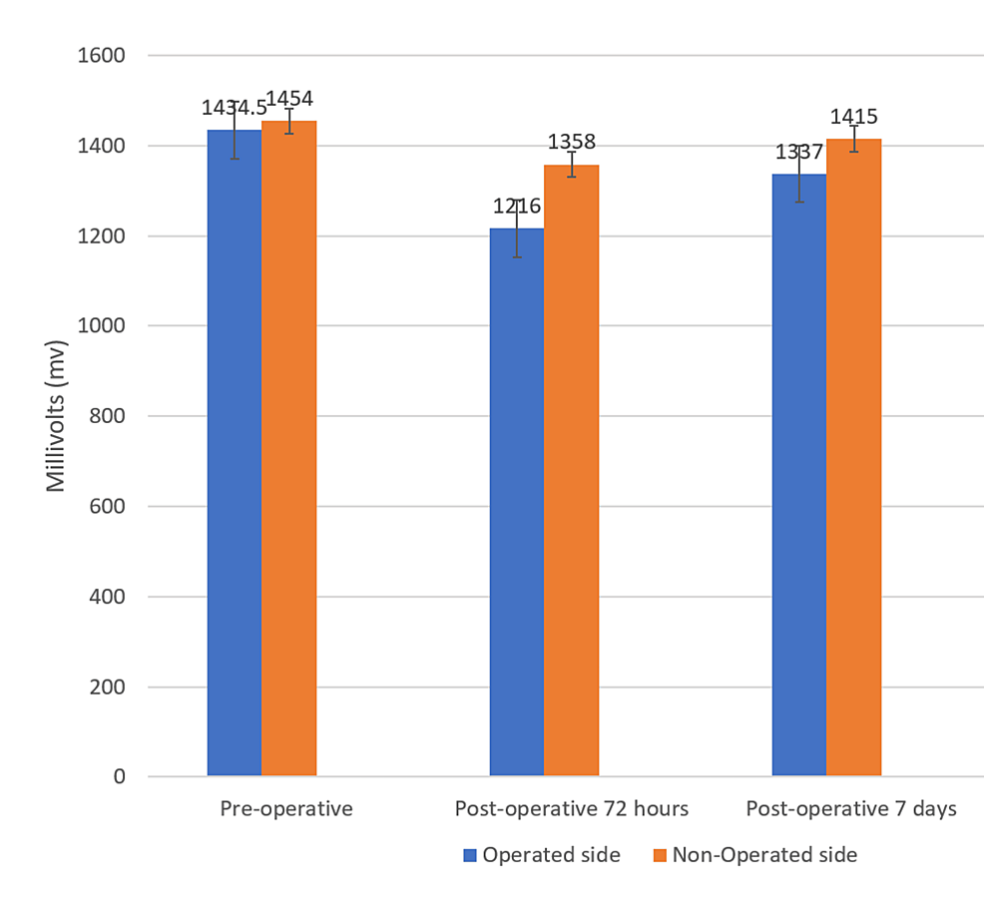
Median Muscle Activity Values Between Operated and Non-Operated Sides for the Temporalis Group at Preoperative, Postoperative 72 hours, and 7 days Postoperative.

A comparison of median values between operated and non-operated sides for the temporalis group at preoperative, postoperative, 72 hours, and 7 days was done. It was found that median electromyographic values were statistically significantly higher on the non-operated side than the operated side in the Temporalis group at preoperative (p = 0.001), postoperative 72 hours (p = 0.001), and postoperative 7 days (p = 0.001) (Figure 4).

## Discussion

There have been many reports in the literature that describe the changes in the masticatory muscle's activity post-lower third molar removal surgery [8-10]. Our study demonstrated the changes in the electrical activity of two major masticatory muscles, i.e., the masseter and the temporalis. One of the most common complications after lower third-molar surgery is postoperative trismus. Despite many newer advances in surgery, this complication still prevails, causing discomfort to the patient [11]. Once the patient gets their lower third molar surgically removed on one side and experiences trismus ranging from either minimal or prolonged duration, they would hesitate to get the other symptomatic side done, keeping in mind their previous experiences [12]. The root cause of trismus has been identified as a change in the dynamics of the masticatory muscles [13]. These muscle-related complications, like trismus, are generally difficult to assess quantitatively [14].

Since EMG can be used to measure the duration, shape of action potentials, and firing frequency of the muscle motor unit, it can also be used to assess masticatory muscle activity. In this study, we have assessed the electrical activity of the masseter and temporalis using surface electrodes. It has been shown in our study that electrical activity has been significantly decreased in patients in both the masseter and temporalis muscle groups [15]. It has been found that, when compared to masseter and temporalis, temporalis recovered faster and returned to its preoperatively measured electrical activity, while it took longer for the masseter to recover on the operated site to return to its original electrical activity [16]. In the existing literature, it has been proven that electrical activity reduction is related to higher trismus activity in patients [17].

Some studies have correlated the reduced electrical activity with clinical signs like maximum mouth opening, and it has been found that in patients with whom there is a faster recovery of the electrical activity to a normal or preoperative value, the recovery is faster concerning the mouth opening and also the reduction of swelling [18]. In this study, it has been found that the electrical activity of the temporalis is higher than that of the masseter muscle measured at all the intervals of the follow-up period, with statistically significant values (p = 0.001). When compared with temporalis, the masseter took longer to return to pre-operative electrical activity, which might also imply that for prolonged trismus seen in patients after lower third molar surgery, it is the masseter that is more affected and needs recovery for the trismus to be resolved [19,20]. 

There have been studies that proved that the use of techniques like low-level laser therapy, which involves photobiomodulation, helps in faster healing and repair of the injured tissues [21,22]. To prevent prolonged trismus in patients’ techniques, low-level laser therapy can be targeted to masseter muscles so that recovery can be accelerated and prolonged trismus can be prevented [23].

Limitations of the study 

More clinical parameters like pain and swelling should have been evaluated and correlated with the electrical activity changes of the masseter and temporalis muscles, and future studies are recommended to assess those parameters. Further studies, including more clinical parameters and a larger sample size, are advised to have better results on this topic. 

## Conclusions

Based on the results obtained, it can be concluded that there was a reduction in the electrical activity of both the masseter and temporalis post-third molar impaction surgery. It was also found that there was a reduction in mouth opening in patients who underwent lower third molar extraction surgery. Masseter muscle took longer to return to its preoperative electrical activity than temporalis muscle, implying that targeted therapies to accelerate the healing of masseter muscle may prevent prolonged trismus in patients who undergo lower third molar impaction surgery.
